# *IFI27* transcription is an early predictor for COVID-19 outcomes, a multi-cohort observational study

**DOI:** 10.3389/fimmu.2022.1060438

**Published:** 2023-01-05

**Authors:** Maryam Shojaei, Amir Shamshirian, James Monkman, Laura Grice, Minh Tran, Chin Wee Tan, Siok Min Teo, Gustavo Rodrigues Rossi, Timothy R. McCulloch, Marek Nalos, Maedeh Raei, Alireza Razavi, Roya Ghasemian, Mobina Gheibi, Fatemeh Roozbeh, Peter D. Sly, Kirsten M. Spann, Keng Yih Chew, Yanshan Zhu, Yao Xia, Timothy J. Wells, Alexandra Cristina Senegaglia, Carmen Lúcia Kuniyoshi, Claudio Luciano Franck, Anna Flavia Ribeiro dos Santos, Lucia de Noronha, Sepideh Motamen, Reza Valadan, Omolbanin Amjadi, Rajan Gogna, Esha Madan, Reza Alizadeh-Navaei, Liliana Lamperti, Felipe Zuñiga, Estefania Nova-Lamperti, Gonzalo Labarca, Ben Knippenberg, Velma Herwanto, Ya Wang, Amy Phu, Tracy Chew, Timothy Kwan, Karan Kim, Sally Teoh, Tiana M. Pelaia, Win Sen Kuan, Yvette Jee, Jon Iredell, Ken O’Byrne, John F. Fraser, Melissa J. Davis, Gabrielle T. Belz, Majid E. Warkiani, Carlos Salomon Gallo, Fernando Souza-Fonseca-Guimaraes, Quan Nguyen, Anthony Mclean, Arutha Kulasinghe, Kirsty R. Short, Benjamin Tang

**Affiliations:** ^1^ Department of Intensive Care Medicine, Nepean Hospital, Penrith, NSW, Australia; ^2^ Centre for Immunology and Allergy Research, the Westmead Institute for Medical Research, Westmead, NSW, Australia; ^3^ Department of Medicine, Sydney Medical School Nepean, Nepean Hospital, University of Sydney, Penrith, NSW, Australia; ^4^ Gastrointestinal Cancer Research Centre, Non-Communicable Diseases Institute, Mazandaran University of Medical Sciences, Sari, Iran; ^5^ Frazer Institute, Faculty of Medicine, The University of Queensland, Brisbane, QLD, Australia; ^6^ Institute for Molecular Bioscience, The University of Queensland, Brisbane, QLD, Australia; ^7^ School of Biomedical Sciences, The University of Queensland, Brisbane, QLD, Australia; ^8^ The Walter and Eliza Hall Institute of Medical Research, Parkville, Melbourne, VIC, Australia; ^9^ Department of Medical Biology, Faculty of Medicine, Dentistry and Health Sciences, University of Melbourne, Melbourne, VIC, Australia; ^10^ Student Research Committee, School of Medicine, Mazandaran University of Medical Sciences, Sari, Iran; ^11^ Antimicrobial Resistance Research Centre, Department of Infectious Diseases, Mazandaran University of Medical Sciences, Sari, Iran; ^12^ Student Research Committee, School of Allied Medical Sciences, Mazandaran University of Medical Science, Sari, Iran; ^13^ Mazandaran University of Medical Sciences, Sari, Iran; ^14^ Child Health Research Centre, The University of Queensland, South Brisbane, QLD, Australia; ^15^ Centre for Immunology and Infection Control, Faculty of Health, School of Biomedical Sciences, Queensland University of Technology, Brisbane, QLD, Australia; ^16^ School of Chemistry and Molecular Biosciences, The University of Queensland, Brisbane, QLD, Australia; ^17^ School of Science, Edith Cowan University; School of Biomedical Science, University of Western Australia, Perth, WA, Australia; ^18^ Complexo Hospital de Clinicas, Universidade Federal do Paraná, Curitiba, Brazil; ^19^ Core for Cell Technology, School of Medicine, PontifìciaUniversidade Católica do Paraná, Curitiba, Brazil; ^20^ Pontifical Catholic University of Parana, Curitiba, Brazil; ^21^ Department of Medical Biotechnology, Faculty of Medical Sciences, Tarbiat Modares University, Tehran, Iran; ^22^ Molecular and Cell Biology Research Centre, Faculty of Medicine, Mazandaran University of Medical Sciences, Sari, Iran; ^23^ Department of Immunology, Faculty of Medicine, Mazandaran University of Medical Sciences, Sari, Iran; ^24^ Biotech Research and Innovation Centre (BRIC), University of Copenhagen, Copenhagen, Denmark; ^25^ Novo Nordisk Foundation centre for Stem Cell Biology, DanStem, Faculty of Health and Medical Sciences, Champalimaud Centre for the Unknown, Lisbon, Portugal; ^26^ Campania Centre for the Unknown, Lisbon, Portugal; ^27^ Department of Clinical Biochemistry and Immunology, Faculty of Pharmacy, University of Concepcion, Concepcion, Chile; ^28^ Molecular and Translational Immunology Laboratory, Department of Clinical Biochemistry and Immunology, Faculty of Pharmacy, Universidad de Concepcion, Concepcion, Chile; ^29^ Faculty of Medicine, Universidad de Concepcion, Concepcion, Chile; ^30^ Infectious Diseases Department, Royal Darwin Hospital, Darwin, NT, Australia; ^31^ Faculty of Medicine, Universitas Tarumanagara, Jakarta, Indonesia; ^32^ Westmead Clinical School, Sydney Medical School, University of Sydney, Sydney, NSW, Australia; ^33^ Sydney Informatics Hub, Core Research Facilities, University of Sydney, Sydney, NSW, Australia; ^34^ Emergency Medicine Department, National University Hospital, National University Health System, Singapore, Singapore; ^35^ Department of Surgery, Yong Loo Lin School of Medicine, National University of Singapore, Singapore, Singapore; ^36^ Faculty of Medicine and Health, School of Medical Sciences, University of Sydney, Sydney, NSW, Australia; ^37^ Centre for Infectious Diseases and Microbiology, Westmead Institute for Medical Research, Sydney, NSW, Australia; ^38^ Westmead Hospital, Western Sydney Local Health District, Sydney, NSW, Australia; ^39^ Queensland University of Technology, Centre for Genomics and PersonalisedHealth, School of Biomedical Sciences, Brisbane, QLD, Australia; ^40^ Critical Care Research Group, The University of Queensland, Brisbane, QLD, Australia; ^41^ Department of Clinical Pathology, Faculty of Medicine, Dentistry and Health Sciences, University of Melbourne, Melbourne, VIC, Australia; ^42^ Australia Centre for Health Technologies (CHT) & Institute for Biomedical Materials & Devices (IBMD), School of Biomedical Engineering, University of Technology Sydney, Sydney, NSW, Australia; ^43^ Exosome Biology Laboratory, Centre for Clinical Diagnostics, University of Queensland Centre for Clinical Research, Royal Brisbane and Women’s Hospital, The University of Queensland, Brisbane, QLD, Australia

**Keywords:** biomarkers, IFI27, SARS-CoV-2, COVID-19, early predictor

## Abstract

**Purpose:**

Robust biomarkers that predict disease outcomes amongst COVID-19 patients are necessary for both patient triage and resource prioritisation. Numerous candidate biomarkers have been proposed for COVID-19. However, at present, there is no consensus on the best diagnostic approach to predict outcomes in infected patients. Moreover, it is not clear whether such tools would apply to other potentially pandemic pathogens and therefore of use as stockpile for future pandemic preparedness.

**Methods:**

We conducted a multi-cohort observational study to investigate the biology and the prognostic role of interferon alpha-inducible protein 27 (*IFI27*) in COVID-19 patients.

**Results:**

We show that *IFI27* is expressed in the respiratory tract of COVID-19 patients and elevated *IFI27* expression in the lower respiratory tract is associated with the presence of a high viral load. We further demonstrate that the systemic host response, as measured by blood *IFI27* expression, is associated with COVID-19 infection. For clinical outcome prediction (e.g., respiratory failure), *IFI27* expression displays a high sensitivity (0.95) and specificity (0.83), outperforming other known predictors of COVID-19 outcomes. Furthermore, *IFI27* is upregulated in the blood of infected patients in response to other respiratory viruses. For example, in the pandemic H1N1/09 influenza virus infection, *IFI27-*like genes were highly upregulated in the blood samples of severely infected patients.

**Conclusion:**

These data suggest that prognostic biomarkers targeting the family of *IFI27* genes could potentially supplement conventional diagnostic tools in future virus pandemics, independent of whether such pandemics are caused by a coronavirus, an influenza virus or another as yet-to-be discovered respiratory virus.

## Introduction

SARS-CoV-2 causes a broad range of clinical outcomes. What determines whether an infected individual will progress to severe COVID-19 is a complex interaction between host and viral factors. Since the beginning of the COVID-19 pandemic, there has been significant interest in developing robust biomarkers to predict disease outcomes amongst COVID-19 patients. This facilitates patient triage and resource prioritisation, both of which become particularly important when healthcare centres are required to simultaneously treat many COVID-19 patients. Numerous prognostic indicators have been proposed for COVID-19. These include, but are not limited to, patient demographics, co-morbidities, lung computed tomography (CT) results, coagulation assays, alterations in white blood cell counts and inflammatory response biomarkers such as C-reactive protein and cytokines (1). Others have used machine learning to predict disease outcomes (2, 3). Despite these investigations, there is no consensus on the best approach for predicting COVID-19 outcomes. Moreover, as there is no indication as to whether such predictive tools would apply to other potentially lethal viruses, and therefore could become part of stockpile for future pandemic preparedness.

We have previously discovered that the transcription of the interferon alpha-inducible protein 27 (*IFI27*) is a signature marker of pandemic H1N1/09 influenza infection (4). *IFI27* (also known as ISG12 or p27) is an interferon alpha (and to a lesser extent interferon gamma) inducible gene of unknown function, with the gene product residing in the nuclear membrane of the cell (5). *IFI27* expression is associated with the outcomes of several different viral illnesses including respiratory syncytial virus (RSV) infection and Enterovirus 71 (EV71) hand foot and mouth disease (6, 7). Preliminary evidence suggests that *IFI27* expression may be a useful biomarker for COVID-19 diagnosis (8–11). In the respiratory tract, *IFI27* expression is significantly upregulated by SARS-CoV-2 infection, more so than by other respiratory viruses (8, 9, 12). *IFI27* is also upregulated in the peripheral blood of COVID-19 patients (10, 11, 13) and may serve as a biomarker for pre-symptomatic SARS-CoV-2 infection (14). These data are consistent with observations that the induction of type I interferons in the lower respiratory tract and systemically is associated with immunopathology (15) whilst the induction of type I interferons in the upper respiratory tract is associated with virus control and not disease outcomes (12). However, others have suggested that IFI27 expression is low in patients with severe COVID (16, 17). Therefore, the usefulness of *IFI27* expression as a prognostic biomarker for COVID-19 has yet to be determined.

Here, we use multiple patient cohorts to evaluate the role of *IFI27* expression in predicting COVID-19 disease progression. We further examine the role of *IFI27* expression for risk stratification in infections caused by other respiratory viruses, namely influenza virus, another virus of pandemic potential.

## Materials and methods

### Patient cohorts

Since *IFI27* is expressed in humans to varying degrees depending on the severity of COVID-19 infection, we assembled multiple patient cohorts (Cohort 1 - 8) to capture the full spectrum of clinical severity (‘Mild’, ‘Moderate’, ‘Severe’ as per definitions below). To ensure that population heterogeneity was adequately represented in the study, we recruited study participants across different countries (Brazil, Iran, Chile, Australia, Singapore, and the U.S.A.). To fully understand *IFI27* expression in different tissue compartments, we systematically evaluated samples of different tissues including lung, upper respiratory tract, and blood. A summary of the relevant cohort characteristics and sampling methods is provided in **Table 1**.

**Table 1:** Overview of cohorts included in the study.

	Country	Year	Study design	Pathogen identified	Time of sampling	SARS-CoV-2 patients recruited	Healthy controls	Gender (males/ females)	Age (Mean years)	Tissue type	Experiment performed	*IFI27* measurement	Clinical setting	Disease severity^@^	Admission to hospital (%)	Death (%)
Cohort 1	Brazil	2020	Case-control study	SARS-CoV-2	Autopsy sample	4	0	2/2	74.5	Lung	RNAscope & Spatial transcriptomics	10X Genomics Visium	Hospital	Severe	4 (100%)	4 (100%)
Cohort 2	Chile	2020	Cross-sectional study	SARS-CoV-2	Presentation to hospital (<1 week post symptom onset)	137	0	92/45	46	Nasal Swabs	qPCR#	qPCR	Hospital	Moderate Severe	130 (100%)	NA
Cohort 3	U.S.A.	2020	Cross-sectional study	SARS-CoV-2	Presentation to hospital (<1 week post symptom onset)	60	0	30/30	55	Nasal Swabs	qPCR	qPCR	Outpatient and hospital	Mild Moderate Severe	38 (63%)	4 (6.7%)
Cohort 4	Chile	2020	Cross-sectional study	SARS-CoV-2	Presentation to hospital (<1 week post symptom onset)	127	0	83/44	54	Plasma	ELISA#	ELISA	Hospital	Moderate Severe	127 (100%)	16 (12.5%)
Cohort 5	Brazil	2020	Cross-sectional study	SARS-CoV-2	Presentation to hospital (<1 week post symptom onset)	28	6	18/10	60	Plasma	ELISA	ELISA	Hospital	Mild Moderate Severe	19 (68%)	4 (14%)
Cohort 6	Iran	2020	Cross-sectional study	SARS-CoV-2	Presentation to hospital (<1 week post symptom onset)	16	6	7/9	53	Blood	qPCR	qPCR	Hospital	Asymptomatic, Mild Moderate	12 (75%)	4 (25%)
Cohort 7	Australia & Singapore	2020	Prospective cohort	SARS-CoV-2	Presentation to hospital (<15 days post symptom onset)	44	14	18/26	49	Blood	qPCR	qPCR	Community, outpatient, and hospital	Mild Moderate Severe	24 (55%)	0 (0%)
Cohort 8	Iran	2021	Prospective cohort	SARS-CoV-2 (Delta)	Presentation to hospital (<1 week post symptom onset)	45	5	26/24	42	Blood	qPCR	qPCR	Hospital	Mild, Moderate, Severe	30 (67%)	0(0%)

**@** For disease severity, ‘Mild’ disease is defined as the presence of COVID-19 disease (as confirmed by the admitted clinician) in a patient who does not require hospitalization. ‘Moderate’ disease is defined as the presence of COVID-19 disease in a patient who requires hospitalization but does not need mechanical ventilation. ‘Severe’ disease is defined as the presence of COVID-19 disease in a patient who requires both hospitalizations and mechanical ventilation (usually in intensive care unit). **#** qPCR denotes ‘quantitative polymerase chain reaction’. ELISA denotes ‘enzyme-linked immunosorbent assay’.

### Definition of severity

We adopted a simplified version of the CDC definition of COVID-19 disease severity (17). In this simplified definition, COVID disease was defined as the presence of suspected COVID-19 symptoms (e.g., fever, sore throat) (**Supplementary Table 1**), together with a positive SARS-CoV-2 detection using virus nucleic acid amplification assay (qPCR). ‘Mild’ disease was defined as the presence of COVID-19 disease (as confirmed by the admitting clinician) in a patient who did not require hospitalization. ‘Moderate’ disease was defined as the presence of COVID-19 disease in a patient who required hospitalization. ‘Severe’ disease was defined as the presence of COVID-19 disease in a patient who required mechanical ventilation (in an intensive care unit).

### Study design

We deployed both retrospective and prospective studies to understand the biology of *IFI27* and to evaluate the clinical utility of the *IFI27* biomarker. Cohort 1 (Brazil) was a case-control study in which the biological role of *IFI27* expression in SARS-CoV-2-induced acute lung injury was examined. Cohorts 2-6 (Iran, Chile, Australia, and the U.S.A.) were cross-sectional studies. We used these studies to compare *IFI27* expression across different tissue compartments (plasma, airways, and blood), to determine the most suitable sampling route for *IFI27* measurement. Cohorts 7 and 8 (Australia, Singapore, and Iran) were prospective cohorts used to validate the prognostic performance of the *IFI27* gene-expression biomarker in a real-world setting. **Table 1** provides a summary of all cohorts included in this study. Additional details on patient recruitment, experiments performed, and data collection are also provided in the **Supplementary Methods**.

### Outcome measure

Cohort 7 (Australia & Singapore) was a prospective validation study of *IFI27* in predicting COVID-19 outcomes. As the sample size was small (n=44), a composite outcome was used to evaluate *IFI27* prediction performance. The composite outcome in a COVID-19 patient was defined as, during the 28-day study period, the first occurrence of: (1) any complication as defined by the International Severe Acute Respiratory and Emerging Infection Consortium (ISARIC), such as viral pneumonia, acute respiratory distress syndrome (ARDS) or bacterial pneumonia (**Supplementary Table 1**); or (2) prolonged virus shedding; or (3) ICU admission; or (4) hospital stay > 7 days. A patient with an ‘adverse’ outcome was one in whom a composite outcome occurred within the 28-day study period and a patient with ‘no adverse’ outcome was one in whom a composite outcome had not occurred during the 28-day study period.

### Predictive performance

We assessed *IFI27* predictive performance using the established methods of Metz and Zhou, as implemented in the NCSS statistical software (Utah, U.S.A) (18). Sensitivity, specificity, positive likelihood ratio and negative likelihood ratio were calculated using the previously established cut-off value for *IFI27* (74) (4). For all performance metrics, 95% confidence intervals were calculated based on the Exact (Clopper-Pearson) method (19). For assessing predictive performance, a previously validated cut-off threshold for *IFI27* expression (fold-change of 74) was used (4). For grouping ‘mild’, ‘moderate’ and ‘severe’ COVID-19 patients, clinical criteria (see ‘Definition of severity’) were used for this purpose.

### Primary cell culture

Primary nasal epithelial cells were grown and infected with SARS-CoV-2 according to the **Supplementary Methods**.

### Experiments

#### cDNA synthesis and qPCR

Total RNA was reverse transcribed using a Qscript cDNA SuperMix (QuantaBio). Amplification of *IFI27* was performed using TaqMan gene expression Master Mix on a CFX384 system. Alternatively, RNA was extracted using Nucleozole reagent according to the manufacturer’s instructions, DNA was removed by DNase I (Thermo Fisher Scientific) treatment and 1µg DNA-free RNA was reverse transcribed into cDNA using the High-Capacity cDNA Reverse Transcription Kit (Applied Biosystems) on a Mastercycler Thermocycler (Eppendorf, Hamburg, Germany) according to the manufacturer’s instructions using random primers. Real-time PCR was performed on generated cDNA with SYBER Green (IFI27 FW: FW: ACCTCATCAGCAGTGACCAGT; RV: ACATCATCTTGGCTGCTATGG or IFI27 FW: CGTCCTCCATAGCAGCCAAGAT; RV: ACCCAATGGAGCCCAGGAT GAA). GAPDH (FW: GTCTCCTCTGACTTCAACAGCG; RV: ACCACCCTGTTGCTGTA GCCAA) or HPRT (FW: TCAGGCAGTATAATCCAAAGATGGT; RV: AGTCTGGCTTAT ATCCAACACTTCG) was used as the endogenous control. The delta-ct method was used to calculate the fold change in gene expression.

#### ELISA

Plasma samples, collected from COVID-19 patients were analysed in duplicate using the IFI27 ELISA kit (Aviva Systems Biology, USA). Plasma *IFI27* (pg/ml) was log2 transformed and median centred to evaluate the relationships to clinical outcomes of COVID-19.

#### RNAscope

RNAscope^®^ probes (Advanced Cell Diagnostics, USA) targeting SARS-CoV-2 spike mRNA were used according to manufacturer’s instructions for automation on the Leica Bond RX of formalin-fixed paraffin embedded (FFPE) rapid autopsy lung tissues from COVID-19 patients and controls. Fluorescent images were acquired with Nanostring Mars prototype DSP at 20x.

#### Spatial transcriptomics

FFPE samples were sectioned at 7µm thickness using a microtome and the section was transferred to a water bath at 41°C. The floating section was adhered to the Visium Spatial Gene Expression Slide (10x Genomics, USA) and processed as per manufacturer recommendations.

#### Microarray

The microarray data of GSE101702, which included 107 influenza patients (n=63 moderate and n=44 severe) and 52 healthy controls, was analysed. The clinical characteristics and additional detail of the dataset are previously described (20). We identified the differentially expressed genes (DEGs) by the R package ‘limma’ (21) between moderate influenza and severe influenza samples. Genes with 1 log_2_fold change with adjusted P value < 0.05 (0.05 FDR) value were considered significant. Full details on the above methods are also provided in **Supplementary Methods**.

### Statistical analysis

Data were tested for normality using the Anderson-Darling test. Where data were normally distributed, they were analysed using an unpaired two-tailed student’s t-test or a one-way ANOVA with a Holm-Šídák’s multiple comparisons test. Where data were not normally distributed, they were analysed using the Mann-Whitney U test or a Kruskal-Wallis test with Dunn’s multiple comparison test. The significance was set at p<0.05. All statistical analyses were performed using Prism version 9.0.

## Results

The findings of this study included expression data (gene/protein) generated across eight cohorts across six countries (Australia, U.S.A., Chile, Brazil, Iran, and Singapore). There was one case-control study (Cohort 1), two prospective studies (Cohort 7 and Cohort 8) and five cross-sectional studies (Cohorts 2-6). Patients included in the studies had different disease severities (mild, moderate, and severe). Cohort 8 (Iran) included COVID-19 patients recruited in 2021, who were infected during the Delta wave of the pandemic in Iran. Other COVID-19 cohorts recruited patients in early 2020 (Cohorts 1-7). Several platforms were used to measure *IFI27* gene expression including PCR, microarray, and spatial transcriptomics. **Table 1** provides full details of the *IFI27* measurement methods and tissue sampling approaches in each cohort.

**Figure 1:**
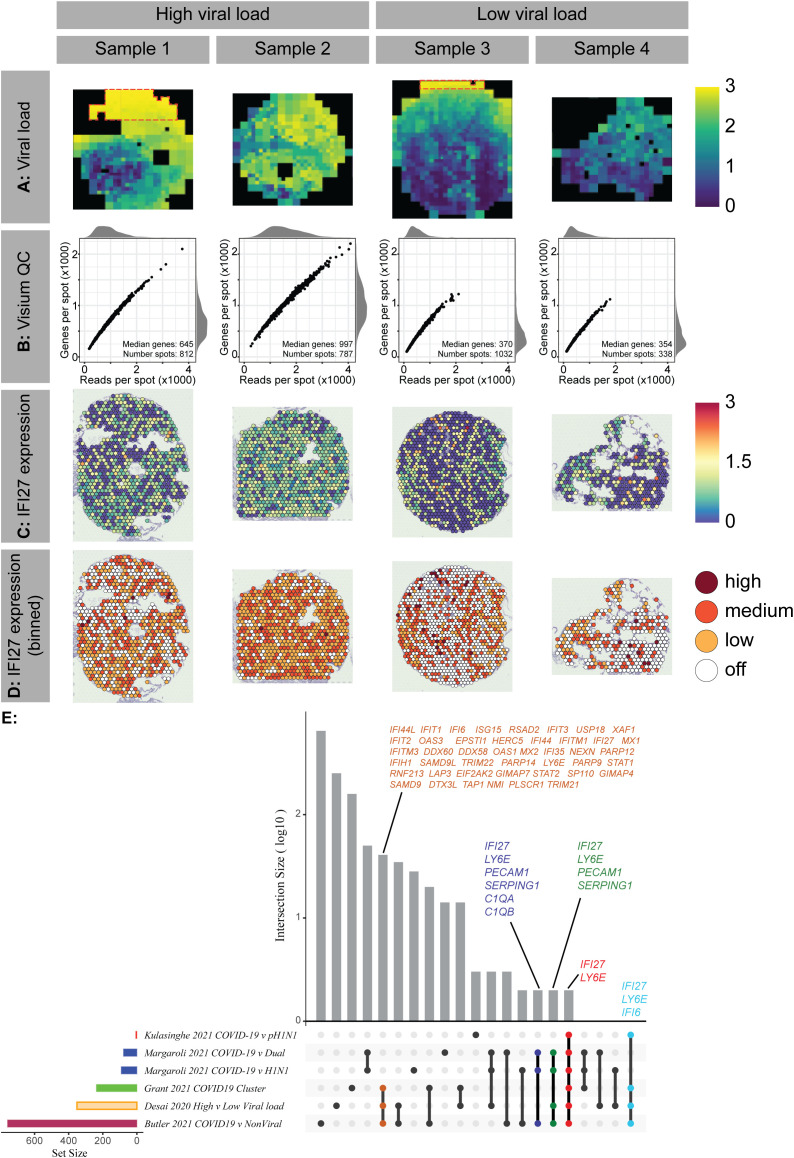
Lower airway IFI27 gene expression in COVID-19 patients A In Cohort 1, spatial expression heatmap of normalised COVID-19 RNAscope signal was determined using STRISH analysis. The heatmap colour shows average RNAscope signal per cell per rectangle area that contains a similar number of cells (fewer than 100 cells per rectangle). Red boxes indicate areas of overexposure in the original RNAscope microscopy, which were excluded from the analysis. B Quality metrics of four multiplexed Visium samples, indicating the number of genes and reads per spot with summary statistics. C Normalised expression of IFI27 across four samples measured in the same Visium experimental slide (poly-A protocol) (blue as low and yellow- red as high). D Binned values of normalised expression of IFI27 across Visium samples. E Upset plot describing the overlapping gene sets across 5 studies. The size of the gene set varies across the studies with a small number of common genes including IFI27 shared across all studies (18-20).

We first investigated whether *IFI27* expression could be detected in COVID-19 patients by assessing gene expression in the lower respiratory tract of deceased COVID-19 patients (Cohort 1; Brazil; n=4). The virus load in the lung was assessed by RNAscope^®^, which had the sensitivity to detect single molecules in a cell. The distribution of virus load was quantified by STRISH, a robust image processing pipeline (22). The average measurements of SARS-CoV-2 spike mRNA (nCoV2019) per grid (tissue region) of neighbouring cells were visualized by STRISH using a tissue heatmap (**Figure 1A**). Adjacent tissue sections from the same samples were then processed by the Visium^®^ spatial transcriptomic method using polyA-capture (**Figure 1B**). Based on comparison of RNAscope and Visium analyses, we discovered that areas of high viral load in the lung, as detected by RNAscope, also had high levels of *IFI27* gene expression (**Figures 1C, D**). The correlation between high viral load and increased *IFI27* gene expression was independently replicated by an additional Visium^®^ spatial dataset, here generated using a probe hybridization protocol (data not shown). Together, these data indicate that *IFI27* expression can be detected in the lower respiratory tract during SARS-CoV-2 infection. This finding was validated by several recently published studies (8, 23, 24), which confirmed the upregulation of *IFI27* expression in infected lung tissue of COVID-19 patients (**Figure 1E**).

**Figure 2:**
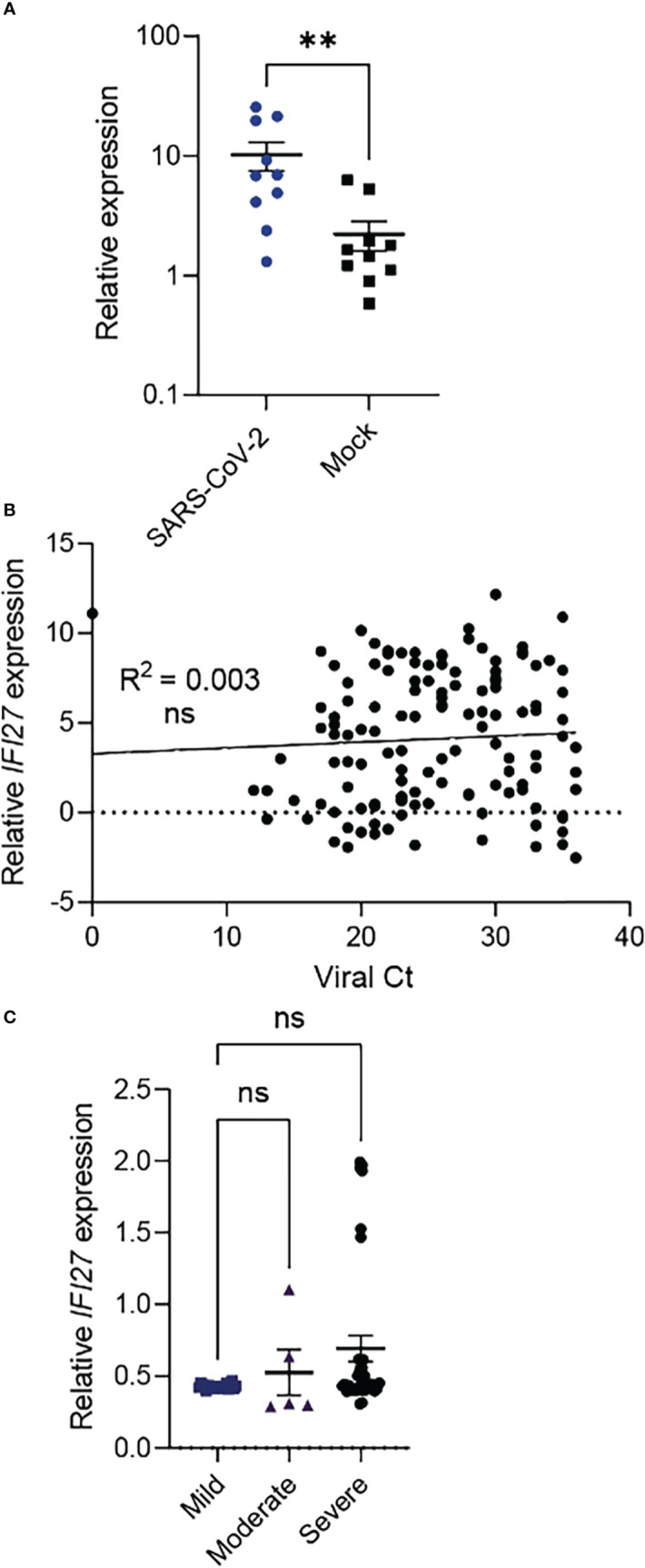
Upper respiratory tract *IFI27* gene expression in COVID-19. **(A)** *IFI27* expression in primary human nasal epithelial cells at 72 hours post-SARS-CoV-2 (QLD/02) or Mock infection. **(B)** *IFI*27 gene expression in nasopharyngeal samples in Cohort 2 (n=137). SARS-CoV-2 virus load (as measured by Ct values) is used as a proxy of local disease activity. A statistically non-significant *p*-value of the linear regression model (represented by R^2^) indicates that there is no association between *IFI*27 expression and viral RNA in the upper respiratory tract. **(C)** *IFI*27 gene expression in nasopharyngeal samples in Cohort 3 (n=60). ‘Mild’ disease is defined as the presence of COVID-19 disease in a patient who does not require hospitalization. ‘Moderate’ disease is defined as the presence of COVID-19 disease in a patient who requires hospitalization. ‘Severe’ disease is defined as the presence of COVID-19 disease in a patient who requires mechanical ventilation in an intensive care unit. p value is calculated using Mann-Whitney U test. **p < 0.01; ns, not significant. Data shows mean ± SEM. *IFI*27 gene expression is measured by qPCR normalized to house-keeping genes.

The above data indicated that *IFI27* gene expression may reflect local disease activity in infected lung tissue. However, lower respiratory tract is not readily accessible or available for prognostic testing. Accordingly, we sought to establish if *IFI27* expression could be detected in the upper respiratory tract during SARS-CoV-2 infection (**Figure 2**). To model the human upper respiratory tract primary nasal epithelial cells from healthy donors were differentiated at an air liquid interface, as described previously (25). Cells were infected with SARS-CoV-2 (QLD02) and *IFI27* expression was assessed 72 hours post-infection. At 72 hours post-infection *IFI27* expression was upregulated in infected nasal epithelial cells (**Figure 2A**). However, the difference in relative expression between infected and uninfected cells (i.e., the dynamic range of expression) was only moderate between infected and uninfected cells (a feature which is suboptimal for a prognostic biomarker). Nevertheless, we further assessed the prognostic potential of *IFI*27 expression in nasopharyngeal swabs of COVID-19 patients (Cohort 2; Chile). Here, no significant association was observed between nasopharyngeal *IFI27* gene expression and virus RNA (**Figure 2B**). Further analysis in another cohort (Cohort 3; USA) showed a similar finding; there was no significant difference in *IFI27* gene expression between mild, moderate, and severe disease (**Figure 2C**). Since these findings suggested that upper airway *IFI27* expression levels do not have a strong prognostic value (and a limited dynamic range) we sought an alternative sampling route to measure *IFI27* expression in COVID-19 patients.

The spread of SARS-CoV-2 infection to the lower airway is often associated with a systemic host response, and blood provides an easily accessible clinical sample to assess this response. Thus, we proceeded to measure *IFI27* expression levels in peripheral blood of COVID-19 patients (Cohort 6; Iran). We found that blood *IFI27* was higher in infected patients (compared to asymptomatic or uninfected individuals). Furthermore, there was a trend of increasing *IFI27* gene expression in patients with a worsening disease, although this trend was not statistically significant (**Supplementary Figure 1A**). When protein expression of *IFI27* was measured, there were no elevation in COVID-19 patients (Cohorts 4 and 5; Chile & Brazil) and *it* did not correlate with disease severity (**Supplementary Figure 1B**). These findings suggested that blood *IFI27* gene expression (not protein expression) could act as a surrogate marker of disease outcome in COVID-19.

**Figure 3:**
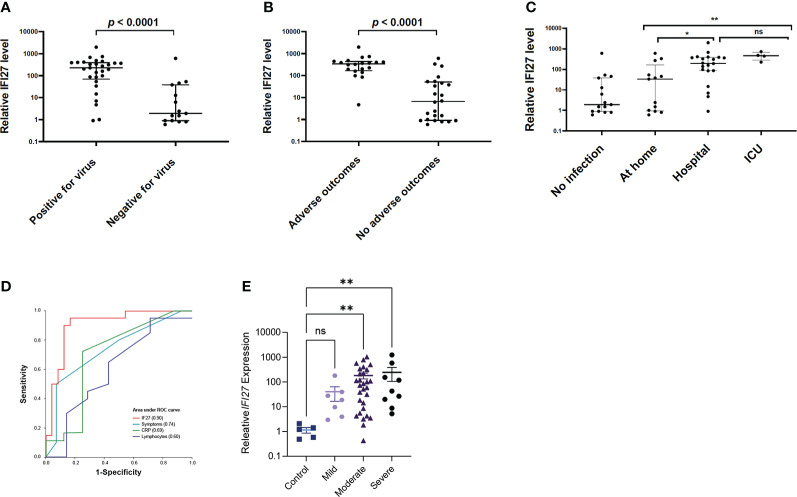
Blood *IFI27* gene expression in COVID-19 patients (prospective cohorts). **(A)** Blood *IFI27* expression in Cohort 7 (n=44), between those patients with a positive SARS-CoV-2 PCR (labelled as ‘positive’) (n=29) and those without (labelled as ‘negative’) (n=15). **(B)** Blood *IFI27* gene expression in Cohort 7 grouped by patients who developed complications (labelled as “adverse outcomes”) (n=20) versus those who did not developed complications (labelled as “no adverse outcomes”) (n=24). **(C)** Blood *IFI27* expression levels grouped by how the patients were managed after infection was diagnosed. A total of 37 patients had adequate follow-up data, including those being managed at home (n=13), admitted to hospital ward (n=20) or admitted to intensive care unit (n=4)”. **(D)** Area-under-the-curve of Receiver-Operator-Characteristics curve (AUROC) analysis of blood IFI27 gene expression levels in predicting composite outcome in Cohort 7 (n=44) *IFI27* gene-expression level (‘IFI27’), total number of symptoms (‘Symptoms’), C-reactive protein (‘CRP’) and lymphocyte count (‘Lymphocytes’). **(E)** *IFI27* expression in Cohort 8, in patients without SARS-CoV-2 (Control) and then those with mild, moderate, and severe disease (as defined in **Table 1**). Data shows mean ± SEM and statistical significance was determined as described in the Methods. *p < 0.05; **p < 0.01; ns, not significant.

We then used an independent prospective cohort (Cohort 7; Australia & Singapore) to test the hypothesis that changes in blood *IFI27* gene expression could predict outcomes in COVID-19 patients. In Cohort 7, blood sampling (for *IFI27* gene expression) was performed upon initial presentation of each patient when the disease outcome was still unknown. Each patient was then followed up for 28 days and their clinical outcomes (e.g., acute respiratory distress syndrome) were recorded (see Methods). We found once again that blood *IFI27* gene expression was significantly higher in patients who tested positive for SARS-CoV-2, a necessary pre-requisite for a prognostic biomarker. (**Figure 3A**) We also noted that *IFI27* blood expression was high in COVID-19 patients who developed an adverse outcome (**Figure 3B**). It is important to emphasise that all patients in this cohort (Cohort 7) were prospectively recruited at the early phase of their infection, which was prior to their outcomes become known (e.g., discharge home or admission to ICU). The patient outcomes were ascertained later in the follow-up period. Therefore, **Figure 3C** demonstrates the predictive capability of blood *IFI27* in the early phase of the infection when the patient’s subsequent trajectory was unknown to the clinician. Since all patients were recruited under the same protocol (i.e., prospective recruitment), the findings in **Figure 3C** reflects the forward performance of *IFI27* of all patients in this cohort. In case studies of a more limited number of patients *IFI27* gene expression increase could precede – by several days – clinical signs of deteriorations or abnormal changes in laboratory parameters such as high levels of C-reactive protein (**Supplementary Figure 2**).

This observation was further confirmed by area under the curve of receiver-operator-characteristics curve (AUROC) analysis, which showed the *IFI27* gene expression outperformed laboratory variables (e.g., C-reactive proteins), patient variables (e.g., age, comorbidity) and physiological parameters (e.g., respiratory rate) in predicting COVID-19 outcomes (**Table 2** and **Figure 3D**). Notably, the *IFI27* gene expression had a high sensitivity (0.95), high specificity (0.83) and an AUROC of 0.90, all of which were higher than known factors associated with COVID-19 outcomes (e.g., age, co-morbidity). To confirm the prognostic value of *IFI27* expression in the blood of COVID-19 patients, a second prospective cohort was recruited (Cohort 8; Iran). Although this cohort was recruited during the peak of the Delta wave in Iran, the same trend was observed: increased *IFI27* expression was observed in patients with more severe COVID-19 (**Figure 3E**).

**Table 2:** Performance of blood *IFI27* gene-expression in predicting composite outcome in Cohort 7.

Parameters	Sensitivity	Specificity	AUROC
***IFI27***	0.95	0.83	0.90*
**Lymphopenia**	0.40	0.71	0.60
**CRP**	0.72	0.75	0.69
**Age**	0.50	0.54	0.57
**Comorbidity**	0.70	0.64	0.77*
**Symptom score**	0.80	0.50	0.74*
**Heart rate^¶^**	0.65	0.73	0.71*
**Respiratory rate^¶^**	0.45	0.91	0.59
**PaO_2_** ¶	0.42	0.64	0.53

AUROC denote area under the curve of receiver-operating-characteristic curve. * Statistically significant (p<0.01).

These measurements (heart rate, respiratory rate and PaO2) were performed on the sample time where blood samples were taken for IFI27 measurement. This is usually done immediately after the patient was recruited into the study (within 24 hours of each patient’s initial presentation). If there were multiple measurements within the same 24 hours period, by convention, the worst reading was recorded.

**Figure 4:**
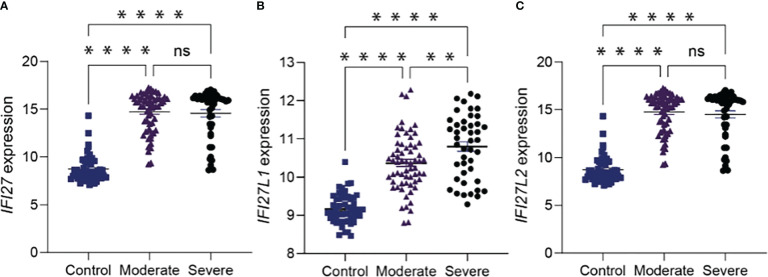
**(A)** *IFI27* like genes have prognostic value in influenza virus infection a *IFI27* expression in the blood of 107 influenza patients.**(B, C)** the *IFI27* gene family; interferon alpha-inducible protein 27-like protein 1 (*IFI27L1*) were best suited to discriminating disease outcome in compared to interferon alpha-inducible protein 27-like protein 2 (*IFI27L2*). ‘Moderate’ disease is defined as the presence of influenza in a patient who requires hospitalization. ‘Severe’ disease is defined as the presence of influenza in a patient who requires mechanical ventilation in an intensive care unit. p value is calculated using Mann-Whitney U test. **p <0.01; ****p<0.0001; ns = not significant. Data shows mean ± SEM.

Ideally, for pandemic preparedness, a prognostic biomarker would function for many viral infections and not be restricted to COVID-19 alone. To investigate this further we analysed blood gene expression data from a previously published microarray study of influenza patients (20). This analysis confirmed that *IFI27* upregulation occurred in severe influenza infection (**Figure 4A**). Importantly, the *IFI27* gene family- including interferon alpha-inducible protein 27-like protein 1 (*IFI27L1*), and not and interferon alpha-inducible protein 27-like protein 2 (*IFI27L2*) (**Figures 4B, C**) were best suited to discriminating disease outcomes. Together, these expanded analyses demonstrated that *IFI27*, or its gene family, could be a prognostic biomarker of the host response to respiratory viruses.

## Discussion

Globally, more than 500K new SARS-CoV-2 infections are recorded every day and the emergence of novel viral variants has raised concerns that these numbers will increase, despite the increasing availability of vaccines. The current global situation emphasises the ongoing need for COVID-19 prognostic biomarkers to facilitate both patient triage and resource prioritisation. Here, we have provided the first evidence of blood *IFI27* expression as a potential biomarker for risk stratification of COVID-19 patients. When prospectively validated, blood *IFI27* expression showed a high positive and negative predictive value, outperforming other known predictors of COVID-19 outcomes reported in the literature.

*IFI27* is an interferon inducible gene. Accordingly, the strong association observed between *IFI27* upregulation and severe COVID-19 could be indicative of an increased viral replication. However, this hypothesis would be inconsistent with the poor correlation observed between nasopharyngeal *IFI27* expression and viral replication in the nose. Instead, we propose that *IFI27* expression reflects increased immunopathology (either local or systemic) and could predict COVID-19 outcome. The correlation of interferon expression and the severity of COVID-19 shows significant anatomical variation. In the upper respiratory tract, rapid induction of type I interferons is typically associated with reduced COVID-19 severity, as their induction is associated with the ability to control viral replication with limited immunopathology (26, 27). In contrast, the relationship between type I interferons and disease pathology is more complex. Certainly, an intact interferon response is important to reduce the severity of SARS-CoV-2 infection. This has been clearly shown by the fact that patients with auto-antibodies to type I IFN (28) or inborn errors in type I IFN immunity (29) experience more severe COVID-19. However, at the same time it is known SARS-CoV-2–infected IFNAR1 KO mice, lacking IFN-α/β signalling, have reduced inflammation and lymphocyte activation, which would support the notion that IFN-α/β can drive pathogenic inflammation in COVID-19 (15). Similarly, patients with severe COVID-19 maintain an elevated interferon response throughout the course of infection, whilst those with moderate disease display a progressive reduction in type I IFN responses over time (30). These seemingly disparate results can be explained by the fact that interferons can play both a protective and pathogenic role during SARS-CoV-2 infection.

What determines whether the interferon response is pathogenic or protective is largely dependent on the magnitude of the response, the kinetics of the response (i.e., if the period of interferon production overlaps with the period of viral replication) and the localisation of the response (i.e., interferon responses in the nose tend to be associated with a more protective response whilst interferon responses in the blood tend to be associated with a more pathogenic response). Importantly, we observed a stronger association between COVID-19 outcome and the *IFI*27 gene expression than that observed with the *IFI27* protein expression in blood. This most likely reflects an increased dynamic range for measuring gene expression and thereby an increased ability to differentiate patient outcomes. Future attempts to translate these findings into routine clinical settings should therefore focus on PCR-based assays to measure *IFI27* expression in blood.

Compared to other biomarkers reported in COVID-19 literature, *IFI27* offers several clinical advantages. Firstly, *IFI27* expression appears to be specific to viral illness. In contrast, most infection/inflammatory biomarkers (e.g., C-reactive protein, leukocytes, interlukin-6) are elevated in many non-viral illnesses (e.g., trauma, sepsis). Secondly, *IFI27* expression is directly linked to intra-cellular recognition of respiratory viruses (15, 26, 27, 30). Given the control point of *IFI27* expression lies in the disease causal pathway, it makes sense to track disease progression by using *IFI27* expression, rather than *via* biomarkers that are unrelated to the underlying disease activity. Finally, blood *IFI27* gene expression has a strikingly high dynamic range (e.g., up to thousands of fold changes in severe COVID-19, as shown in the present study) and a strong signal-to-noise ratio (which produced consistent findings across different measurement platforms, such as microarray or PCR, also evidenced in our findings). These favourable measurement characteristics makes *IFI27* a preferred prognostic tool to other biomarkers.

The kinetics of *IFI27* expression is poorly understood. As a result, the ideal sampling window was not well-defined in this study. It is also uncertain whether serial measurement of *IFI27* expression would be more informative than a single time point measurement. Furthermore, the predictive performance of *IFI27* could be confounded by other yet-to-be-defined variables, such as timing (e.g., early presentation versus late presentation), stages of disease (e.g., lung only infection versus multi-organ disease) and prior COVID-19 vaccination (only measured in Cohort 8 in the present study). Indeed, some studies have suggested *IFI27* expression is lowest in the critical care or ICU COVID-19 patients (16, 31) which is as apparently incongruent with the data presented herein. These differences may reflect the fact some critical cases have known defects in their interferon response (28) or, age differences between cohorts (noting that the mean age for cohort 7 was relatively low). Alternatively, we noted that the two critical patients in the study of Huang et al. were first sampled approximately 14 days after diagnosis (which was markedly later than those in the present study) and these differences may reflect kinetic differences in *IFI27* expression. Therefore, we are unable to extrapolate the prognostic value of blood *IFI27* expression to a broader clinical context and additional studies are required. Such studies should consist of large-scale, prospective studies with sample sizes adequately powered to allow researchers to assess the independent confounding effect of each variable (e.g., timing, disease stages and prevalence) on *IFI27* expression.

The COVID-19 pandemic has emphasised the need to have stockpiles of broad-spectrum diagnostic and therapeutic tools that can be rapidly deployed at the start of any future viral outbreak. As a component of the anti-viral interferon response, it is not surprising that elevated *IFI27* expression was observed in the blood of patients infected with a broad range of different respiratory viruses but not in the blood of patients with a bacterial respiratory infection. These data, combined with previous suggestions that *IFI27* expression is associated with the severity of RSV (6, 7), suggest that *IFI27* may represent a pan-viral prognostic biomarker. Interestingly, when we investigated this further in the context of influenza virus, we found that expression of *IFI27*L1 in the blood, rather than *IFI27* itself, was associated with disease severity. We therefore propose that future studies focus on developing PCR-based assays to measure a suite of genes associated with *IFI27* expression including *IFI27*L1, *IFI27*L2 and IFI6. It is hoped that this broader, combinatorial approach, would lead to the development of pan-viral prognostic biomarker that could be incorporated into future pandemic preparedness planning.

The basis for ancestral differences in IFN expression is not well-explained. A study shows that in SLE patients a cluster of highly expressed ISGs including *IFI27* is more associated with African ancestry than disease activity (32). Our study which combines cohorts from ethnically diverse populations (Middle Eastern, Asian, European & South American) shows robust nature of IFI27 as a prognostic biomarker.

The present study has several limitations. Firstly, the prospective validation cohorts were relatively limited in size. Secondly, most patient cohorts (Cohort 1-7) were recruited prior to the availability of vaccination. One cohort (Cohort 8) did include vaccinated individuals recruited during the Delta wave of COVID-19 in Iran. Accordingly, our findings do not necessarily apply to vaccinated individuals experiencing ‘breakthrough’ Omicron (or subsequent variant) infections. Finally, we could not correlate the changes in *IFI27* expression levels between the peripheral blood and the infected lung. Future studies (e.g., in animal models) are needed to unravel the coupled dynamics of *IFI27* expression between these tissue compartments.

## Conclusions

The findings provided herein represent the first evidence that *IFI27* expression has a potential as a blood biomarker for risk stratification in COVID-19 patients.

## Data availability statement

The original contributions presented in the study are included in the article/**Supplementary Material**. Further inquiries can be directed to the corresponding authors.

## Ethics statement

The study was approved under the Human Research Ethics Committee at Western Sydney Local Health District (HREC Reference: 2020/ETH00886 (6439), Research Governance at Westmead Institute for Medical Research, and Nepean Blue Mountain Local Health District (HREC Reference: 2019/ETH01485. Autopsy and biopsy materials were obtained from the Pontificia Universidade Catolica do Parana PUCPR the National Commission for Research Ethics (CONEP) under ethics committee approval reference number 2020001792/30188020.7.1001.0020 and approval reference number 2020001934/30822820.8.000.0020. University of Queensland Human Research Ethics Committee (HREC) ratification. Human Research Ethics Committees of the University of Concepcion (Chile) (CEBB 676-2020) and ratified by the University of Queensland (2021/HE000319). Human Research Ethics Committee of the Pontifícia Universidade Católica do Paraná (PUCPR) (Number CAAE: 30833820.8.0000.0020) and by Comissão Nacional de Ética em Pesquisa (CONEP). Mazandaran University of Medical Sciences (approval number IR.MAZUMS.REC 1399.856). National Healthcare Group Domain Specific Review Board (DSRB 2014/00614). The University of Queensland’s Human Research Ethics Committee (2020001742), the Queensland Children’s Hospital and Health Service Human Research Ethics Committee (HREC/16/QRCH/215, HREC/10/QRCH/78) and the Queensland University of Technology Human Research Ethics Committee 17000000039). All studies with SARS-CoV-2 were performed under physical containment 3 (PC3) conditions and were approved by the University of Queensland Biosafety Committee (IBC/374B/SCMB/2020). Written informed consent was obtained from all study participants. All methods were performed in accordance with institutional guidelines and regulations.

## Author contributions

Conceptualisation: AK, MS, KS, BT, AM. Investigation: AS, JM, LC, MT, CT, SMT, GR, TM, MN, MR, AR, RoG, MG, FR, PS, KS, KC, YZ,YX, TW, ACS, CK, CF, AFRS, LN, SM, RV, OA, RaG, EM, RA-N, LL, FZ, EN-L, GL, BK, VH, YW, AP, TC, TK, KK, ST, TP, TK, TJ, JI, KO’B, JF, MW, CG, FS-F-G. Formal analysis: MS, KS, BT, AK, CT, RoG, YW, KO’B, JF, MD, GB, QN. Writing - original draft: all authors. Writing - review & editing: all authors. All authors contributed to the article and approved the submitted version.

## References

[1] Gallo Marin B Aghagoli G Lavine K Yang L Siff EJ Chiang SS. Predictors of COVID-19 severity: A literature review. Rev Med Virol (2021) 31(1):1–10. doi: 10.1002/rmv.2146 PMC7855377 32845042

[2] Chowdhury ME Rahman T Khandakar A Al-Madeed S Zughaier SM Doi SA. An early warning tool for predicting mortality risk of COVID-19 patients using machine learning. Cogn Comput (2021), 1–16. doi: 10.1007/s12559-020-09812-7 PMC8058759 33897907

[3] Shu T Ning W Wu D Xu J Han Q Huang M. Plasma proteomics identify biomarkers and pathogenesis of COVID-19. Immunity (2020) 53(5):1108–22.e5. doi: 10.1016/j.immuni.2020.10.008 33128875 PMC7574896

[4] Tang BM Shojaei M Parnell GP Huang S Nalos M Teoh S. A novel immune biomarker IFI27 discriminates between influenza and bacteria in patients with suspected respiratory infection. Eur Respir J (2017) 49(6). doi: 10.1183/13993003.02098-2016 28619954

[5] Martensen PM Søgaard TMM Gjermandsen IM Buttenschøn HN Rossing AB Bonnevie-Nielsen V. The interferon alpha induced protein ISG12 is localized to the nuclear membrane. Eur J Biochem (2001) 268(22):5947–54. doi: 10.1046/j.0014-2956.2001.02545.x 11722583

[6] Min Z Ye Z Gang L Boyu D Xueyan X. IFI27 as a potential indicator for severe enterovirus 71-infected hand foot and mouth disease. Virus Res (2020) 289:198149. doi: 10.1016/j.virusres.2020.198149 32866535

[7] Gao J Zhu X Wu M Jiang L Wang F He S. IFI27 may predict and evaluate the severity of respiratory syncytial virus infection in preterm infants. Hereditas (2021) 158(1):1–14. doi: 10.1186/s41065-020-00167-5 33388093 PMC7778825

[8] Kulasinghe A Tan CW dos Santos Miggiolaro AFR Monkman J Bhuva D JdSM J. Spatial profiling of lung SARS-CoV-2 and influenza virus infection dissects virus-specific host responses and gene signatures. medRxiv (2020). doi: 10.1101/2020.11.04.20225557 PMC8542865 34675048

[9] Mick E Kamm J Pisco AO Ratnasiri K Babik JM Calfee CS. Upper airway gene expression differentiates COVID-19 from other acute respiratory illnesses and reveals suppression of innate immune responses by SARS-CoV-2. medRxiv (2020). doi: 10.1101/2020.05.18.20105171

[10] Shaath H Vishnubalaji R Elkord E Alajez NM. Single-cell transcriptome analysis highlights a role for neutrophils and inflammatory macrophages in the pathogenesis of severe COVID-19. Cells (2020) 9(11):2374. doi: 10.3390/cells9112374 33138195 PMC7693119

[11] Huang L Shi Y Gong B Jiang L Liu X Yang J. Blood single cell immune profiling reveals the interferon-MAPK pathway mediated adaptive immune response for COVID-19. MedRxiv (2020). doi: 10.1101/2020.03.15.20033472

[12] Li X Kolling FW Aridgides D Mellinger D Ashare A Jakubzick CV. ScRNA-seq expression of IFI27 and APOC2 identifies four alveolar macrophage superclusters in healthy BALF. Life Sci Alliance (2022) 5(11). doi: 10.26508/lsa.202201458 PMC9275597 35820705

[13] Wang Y Li J Zhang L Sun H-X Zhang Z Xu J. Plasma cell-free RNA characteristics in COVID-19 patients. Genome Res (2022). doi: 10.1101/gr.276175.121 PMC8805721 35064006

[14] Gupta RK Rosenheim J Bell LC Chandran A Guerra-Assuncao JA Pollara G. Blood transcriptional biomarkers of acute viral infection for detection of pre-symptomatic SARS-CoV 2 infection. medRxiv (2021). doi: 10.1101/2021.01.18.21250044 PMC8260104 34250515

[15] Israelow B Song E Mao T Lu P Meir A Liu F. Mouse model of SARS-CoV-2 reveals inflammatory role of type I interferon signaling. J Exp Med (2020) 217(12). doi: 10.1084/jem.20201241 PMC7401025 32750141

[16] Overmyer KA Shishkova E Miller IJ Balnis J Bernstein MN Peters-Clarke TM. Large-Scale multi-omic analysis of COVID-19 severity. Cell Syst (2021) 12(1):23–40.e7. doi: 10.1016/j.cels.2020.10.003 33096026 PMC7543711

[17] Available at: https://www.covid19treatmentguidelines.nih.gov/overview/clinical-spectrum.

[18] Metz CE. editor Basic principles of ROC analysis. Seminars in nuclear medicine (1978) 4(8):283–298. 10.1016/s0001-2998(78)80014-2 112681

[19] Upton G Cook I. (2014). A dictionary of statistics 3e: Oxford university press.

[20] Zerbib Y Jenkins EK Shojaei M Meyers AF Ho J Ball TB. Pathway mapping of leukocyte transcriptome in influenza patients reveals distinct pathogenic mechanisms associated with progression to severe infection. BMC Med Genomics (2020) 13(1):1–13. doi: 10.1186/s12920-020-0672-7 32066441 PMC7027223

[21] Ritchie ME Phipson B Wu D Hu Y Law CW Shi W. Limma powers differential expression analyses for RNA-sequencing and microarray studies. Nucleic Acids Res (2015) 43(7):e47–e. doi: 10.1093/nar/gkv007 PMC4402510 25605792

[22] Tran M Yoon S Teoh M Andersen S Lam PY Purdue BW. A robust experimental and computational analysis framework at multiple resolutions, modalities and coverages. Front Immunol (2022) 13:911873. doi: 10.3389/fimmu.2022.911873 35967449 PMC9373800

[23] Margaroli C Benson P Sharma NS Madison MC Robison SW Arora N. Spatial mapping of SARS-CoV-2 and H1N1 lung injury identifies differential transcriptional signatures. Cell Rep Med (2021) 2(4):100242. doi: 10.1016/j.xcrm.2021.100242 33778787 PMC7985929

[24] Grant RA Morales-Nebreda L Markov NS Swaminathan S Querrey M Guzman ER. Circuits between infected macrophages and T cells in SARS-CoV-2 pneumonia. Nature (2021) 590(7847):635–41. doi: 10.1038/s41586-020-03148-w PMC7987233 33429418

[25] Zhu Y Chew KY Karawita AC Yamamoto A Labzin LL Yarlagadda T. Pediatric nasal epithelial cells are less permissive to SARS-CoV-2 replication compared to adult cells. bioRxiv (2021). doi: 10.1101/2021.03.08.434300

[26] Blanco-Melo D Nilsson-Payant BE Liu WC Uhl S Hoagland D Møller R. Imbalanced host response to SARS-CoV-2 drives development of COVID-19. Cell (2020) 181(5):1036–45.e9. doi: 10.1016/j.cell.2020.04.026 32416070 PMC7227586

[27] Hoagland DA Møller R Uhl SA Oishi K Frere J Golynker I. Leveraging the antiviral type I interferon system as a first line of defense against SARS-CoV-2 pathogenicity. Immunity (2021) 54(3):557–70.e5. doi: 10.1016/j.immuni.2021.01.017 33577760 PMC7846242

[28] Bastard P Rosen LB Zhang Q Michailidis E Hoffmann HH Zhang Y. Autoantibodies against type I IFNs in patients with life-threatening COVID-19. Science (2020) 370(6515). doi: 10.1126/science.abd4585 PMC7857397 32972996

[29] Zhang Q Bastard P Liu Z Le Pen J Moncada-Velez M Chen J. Inborn errors of type I IFN immunity in patients with life-threatening COVID-19. Science (2020) 370(6515):eabd4570. doi: 10.1126/science.abd4570 32972995 PMC7857407

[30] Lucas C Wong P Klein J Castro TBR Silva J Sundaram M. Longitudinal analyses reveal immunological misfiring in severe COVID-19. Nature (2020) 584(7821):463–9. doi: 10.1038/s41586-020-2588-y PMC7477538 32717743

[31] Huang L Shi Y Gong B Jiang L Zhang Z Liu X. Dynamic blood single-cell immune responses in patients with COVID-19. Signal transduction targeted Ther (2021) 6(1):1–12. doi: 10.1038/s41392-021-00526-2 PMC7936231 33677468

[32] Siddiqi KZ Wilhelm TR Ulff-Møller CJ Jacobsen S. Cluster of highly expressed interferon-stimulated genes associate more with African ancestry than disease activity in patients with systemic lupus erythematosus. A systematic review of cross-sectional studies. Transl Res (2021) 238:63–75. doi: 10.1016/j.trsl.2021.07.006 34343626

