# Supplementary figures and images for: IFI27 transcription is an early predictor for COVID-19 outcomes, a multi-cohort observational study
Source: Front Immunol. 2023 Jan 5;13:1060438. doi: 10.3389/fimmu.2022.1060438 (PMC9850159; doi:10.3389/fimmu.2022.1060438)

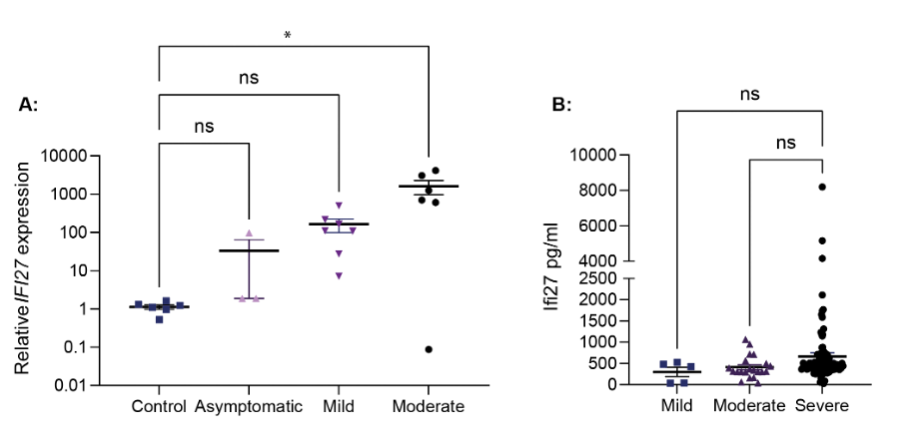

Supplement: Supplementary file 1 [file Image_1.tiff]

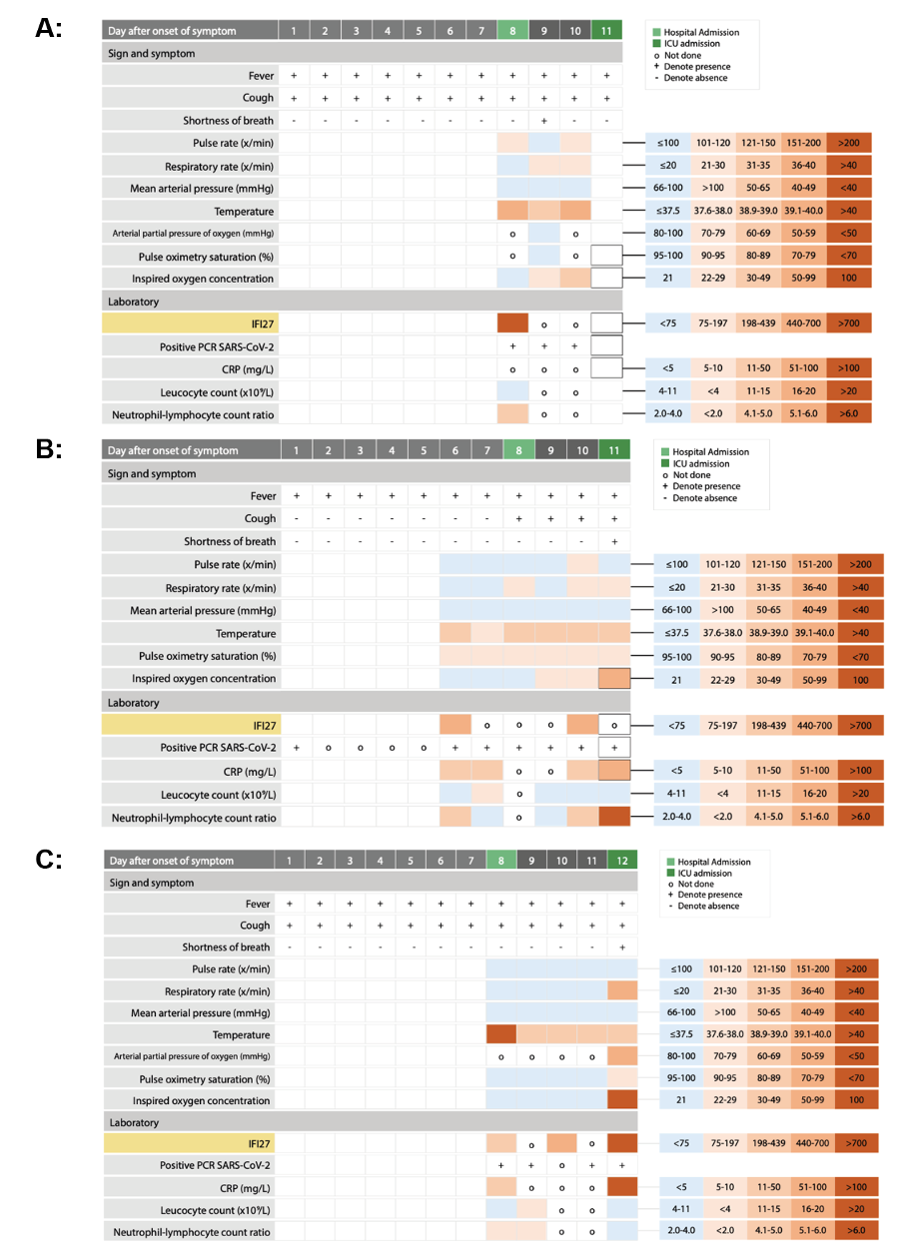

Supplement: Supplementary file 2 [file Image_2.tiff]
