# Supplementary material for: IFI27 transcription is an early predictor for COVID-19 outcomes, a multi-cohort observational study
Source: Front Immunol. 2023 Jan 5;13:1060438. doi: 10.3389/fimmu.2022.1060438 (PMC9850159; doi:10.3389/fimmu.2022.1060438)
Supplement: Supplementary file 3 [file DataSheet_1.docx]

**Supplementary Information**

**This file includes:**

Supplementary Methods

Table 1 Legend

Figure 1 Legend

Figure 2 Legend

**Other Supplementary Information includes the following (separate file):**

Supplementary Table 1

Supplementary Figure 1

Supplementary Figure 2

**Supplementary Methods**

**Cohort 1**

Tissue microarray cores were prepared from autopsied pulmonary tissue from SARS-CoV-2 patients who died from respiratory failure (ARDS). All SARS-CoV-2 patients were confirmed for infection through RTqPCR of nasopharyngeal swab specimens, and imaging with computed tomography (CT) showed diffuse and bilateral opacities with ground-glass attenuation, suggestive of viral pulmonary infection.

**Cohort 2 & 4**

Study participants were recruited from Hospital Regional by Dr. Guillermo Grant Benavente, Concepcion, Chile. The inclusion criteria were nasopharyngeal swab RT-PCR confirmed SARS-CoV-2 infection. Nasal samples (n=137) and blood samples (n=127) were collected. Nasal samples (n=137) were grouped as “Cohort 2” and blood samples (n=127) were grouped as “Cohort 4”. COVID-19 disease severity at presentation to hospital was recorded for the patient group from which the blood samples were obtained. Mild (n=3), Moderate (n=16) and severe (n=108). “Mild” disease is defined as the presence of COVID-19 disease in a patient who does not require hospitalization. “Moderate” disease is defined as the presence of COVID-19 disease in a patient who requires hospitalization. “Severe” disease is defined as the presence of COVID-19 disease in a patient who requires mechanical ventilation in an intensive care unit.

**Cohort 3**

The COVID-19 samples in form of the nasopharyngeal swabs were procured from the TSB BioBank, which is part of the Translational Science BioCore (TSB) affiliated with the UW Carbone Cancer Center (UWCCC), University of Wisconsin-Madison School of Medicine and Public Health, Madison, Wisconsin, USA. Coronavirus Disease 2019 (COVID-19), PCR (UWH) test was performed on nasopharyngeal swabs on Molecular Genprobe Panther Fusion platform in Molecular Diagnostics Lab. Chart review was performed on admitted patients negative for COVID19 and patients who presented to ED with symptoms consistent with COVID19 infection, such as fever, cough, and dyspnea were included in the cohort. Chart review was performed on admitted patients positive for COVID-19 and patients who had only 1 positive COVID-19 test result in their clinical history on the day of their admission were included in the cohort.

**Cohort 5**

Study participants were recruited from Complexo Hospital de Clinicas da Universidade Federal do Paraná who had tested positive by PCR for SARS-CoV-2. Subjects with previous or current history of malignancy; thromboembolic pathology; severe allergic reaction; concomitant infection with HIV, tuberculosis, or other respiratory virus; transplant or use of immunosuppressive therapy and pregnancy or breastfeeding were not included in the study. Patient information is listed in Table 1.

**Cohort 6**

Study participants were individuals with nasopharyngeal swab RT-PCR confirmed SARS-CoV-2 infection and computed tomography (CT) scan with ground-glass opacities. A subgroup of participants without SARS-CoV-2 infection were included as controls. Patients (n=16) and uninfected individuals (n=6) were recruited for this study and provided blood samples at the time of presentation to hospital. Patients were categorized into asymptomatic (n=3), mild (n=7) and moderate (n=6) stages of COVID-19 severity. “Mild” disease is defined as the presence of COVID-19 disease in a patient who does not require hospitalization. “Moderate” disease is defined as the presence of COVID-19 disease in a patient who requires hospitalization. “Severe” disease is defined as the presence of COVID-19 disease in a patient who requires mechanical ventilation in an intensive care unit.

**Cohort 7**

*Singaporean cohort:* Study participants were individuals with suspected respiratory infections during the onset of the COVID-19 pandemic at the National University Hospital, Singapore. Individuals were considered COVID-19 patients if the patient tested positive for the virus by RT-PCR on admission (n=2). *Australia cohort:* Study participants were individuals with suspected respiratory infections during the onset of the COVID-19 pandemic in the Southern Hemisphere (Westmead Hospital, Nepean Hospital, Sydney Australia and National University Hospital, Singapore). Subjects with recent (within the prior 14 days) vaccination history, infection/under antimicrobial medication, subjects under immunosuppressive drugs were not included in the study. Individuals became eligible for the study immediately upon the reporting of suspected COVID-19 symptoms (e.g., fever, sore throat, cough). Individuals were considered COVID-19 patients if the patient tested positive for the virus by RT-PCR on admission or in the subsequent 28-day follow-up period.

**Cohort 8**

Study participants were individuals with nasopharyngeal swab RT-PCR confirmed SARS-CoV-2 infection and computed tomography (CT) scan with ground-glass opacities. A subgroup of participants without SARS-CoV-2 infection were included as controls. Patients (n=45) and uninfected individuals (n=5) were prospectively recruited for this study and provided blood samples at the time of presentation to hospital. Patients were categorized into mild (n=7) and moderate (n=29) and severe (n=9) stages of COVID-19 severity. “Mild” disease is defined as the presence of COVID-19 disease in a patient who does not require hospitalization. “Moderate” disease is defined as the presence of COVID-19 disease in a patient who requires hospitalization. “Severe” disease is defined as the presence of COVID-19 disease in a patient who requires mechanical ventilation in an intensive care unit.

**Experiments performed in Cohort 1**

**Rapid Autopsy Tissue**

Formalin-fixed paraffin-embedded (FFPE) tissue blocks were prepared from autopsied pulmonary tissue from 4 COVID-19 patients who died from respiratory failure (ARDS). Details of the patient cohort have been described previously (1).

**RNAscope^®^ of lung tissue**

RNAscope^®^ probes (ACDbio, US) targeting SARS-CoV-2 spike mRNA (nCoV2019, #848561-C3) were used as per manufacturer instructions for automation on Leica Bond RX. DNA was visualised with Syto13 (Thermofisher Scientific). Fluorescent images were acquired with Nanostring Mars prototype DSP at 20x. RNAscope data from NanoString was analysed by the STRISH program (Tran et al, 2020). STRISH first performed cell segmentation and followed by mapping the SARS-CoV-2-positive cells based on RNAscope fluorescent signal (nCoV2019 spike mRNA). Subsequently, the software scanned through the whole tissue and counted cells with positive SARS-CoV-2 marker within segmented cells. STRISH gradually split the image into smaller windows until each window contains fewer than 100 cells. STRISH then performed min-max normalisation and plotted a heatmap to display the level of expression signal. The analysis code is available at the following weblink:

<https://github.com/BiomedicalMachineLearning/Covid19>.

**Spatial transcriptomics (Visium)**

PolyA-capture: FFPE tissue blocks were sectioned at 7μm thickness using a microtome and the section was transferred to a water bath at 41°C. The floating section was adhered to the Visium Spatial Gene Expression Slide (10x Genomics, USA, PN 2000233), and stored overnight at 4°C. The slide was deparaffinised by placing it on the surface of a thermomixer at 60°C for 30 minutes, immersing in Xylene for 5 mins twice and in ethanol six times, 2 minutes each. The H&E staining was carried out as per the Visium instructions, except that 100μl of glycerol 85% was used. Tissue imaging was performed using an Axio Scan Z1 Fluorescent Slide Scanner. After H&E imaging, the slide went through pre-permeabilization (37°C, with collagenase mix for 20 mins), decrosslinking (70°C for 1 hour, in TE buffer pH 8.0) and permeabilization (75μl 0.1% Pepsin, 37°C for 10 minutes). Library preparation was performed according to the Visium Gene Expression user guide (CG000239). The Visium raw sequencing data in BCL format was converted to 137,604,799 FASTQ reads using bcl2fastq/2.17. The reads were trimmed by cutadapt/1.8.3 to remove poly-A tails and template-switching-oligos (slide V10N16-049-B1). We used SpaceRanger V1.2.2 to map FASTQ reads to the CellRanger human reference genome and gene annotation for GRCh38-3.0.0. On average, for each spot we mapped 45,914 reads and detected a total of 17,598 genes, with an average of 565 genes per spot. We applied an adaptive binning strategy for the visualisation of gene expression for individual spots across all tissues. Four bins were calculated: off, low, medium, and high expression. The low to high bins were defined separately for each gene based on equal division of their normalised expression; genes with no counts were added to the off bin.

**ELISA performed in Cohorts 4 & 5**

A volume of 4.0ml of whole blood was collected in EDTA tubes (BD Frankilin Lakes, NJ, USA). The tubes were centrifuged for 10 minutes at 400g, and the plasma obtained was stored at -80°C for long term storage. 25ul of plasma from these patients was analysed in duplicate using IFI27 ELISA kit (Aviva Systems Biology, USA). Serum IFI27 (pg/ml) were log_2_ transformed and median centred to evaluate the relationship with clinical annotation.

**Primary nasal epithelial cells**

Primary nasal epithelial cells were collected from healthy individuals (aged 2-37 years old) donors by placing a sterile nasal mucosal curette (Arlington Scientific Inc., USA) in the mid-inferior portion of the inferior turbinate. Primary NECs were established *in vitro* as previously described (2-4) and stored in freezing media (FBS with 10% DMSO) at passage 1 or 2. In total, we included 10 donors in this study (adult aged 28.2±7.3 years old, N = 5 (4 females and 1 males) and pediatric aged 4.6±1.8 years old, N=5 (2 female, 3 male).

**Viral infection**

SARS-CoV-2 isolate hCoV-19/Australia/QLD02/2020 (QLD02) was kindly provided by Queensland Health Forensic & Scientific Services, Queensland Department of Health and the Kirby Institute. Virus was amplified in Vero cells expressing human TMPRSS2 and titrated by plaque assay (5). Differentiated nasal epithelial cells were infected with mock (PBS) or QLD02 (1.25 x 10^5^ PFU). Specifically, 100uL of virus or PBS was placed on the epithelial surface in the apical compartment and incubated for 1 hour at 37ºC. Following incubation, excess virus was removed from the transwell and cells were incubated at 37ºC with 5% CO_2_. Every 24 hours the basolateral media was refreshed with 1mL of new ALI media. At 72 hours post-infection cells were lysed with Buffer RLT (Qiagen, USA) containing 0.01% β-mercaptoethanol for RNA analysis.

**Supplementary Table 1 legend**

Simplified version of the CDC definition of COVID-19 disease severity

**Supplementary Figure 1 legend**

Blood *IFI27* gene expression in COVID-19 patients (Cross-sectional studies) **A** Blood *IFI27* gene expression in Cohort 6 (n = 22). Disease severity as defined in Table S1. **B** Blood *IFI27* protein-expression levels in Cohorts 4 and 5 (n=161). *IFI*27 protein-expression and statistical significance was determined as described in the Methods. (*p <0.05, ns = not significant).

**Supplementary Figure 2 legend**

Three representative patients (A, B and C) from Cohort 7 are presented here:

1. The clinical trajectory of each patient is plotted along a time-axis (in days), which is located at the top of the graph. Along this axis, the day of hospital admission or ICU admission is indicated by green colour.
2. Within each graph, a plus or minus sign (“+” or “-”) indicates the presence or absence of an observation (e.g. fever) or a measurement (e.g. positive SARS-CoV-2 virus). If an observation or measurement was not performed, then an “o” sign is provided.
3. A colour scale (located on the right-hand side of the graph) is used to indicate the degree of abnormality in each observation or measurement. Blue colour indicates the observation or measurement is within the normal range. Light red colour indicates a mildly abnormal observation or measurement. Deep red colour indicates a highly abnormal observation or measurement.
4. The *IFI27* gene expression is expressed as fold change.

**Figure 2 legend (continue)**

**Patient A**

*History and physical examination*

This patient presented with 7 days of fever and cough. On the day of hospital admission, the patient had a mildly elevated heart rate (101 – 120/min), but other vital signs were normal, including respiratory rate (< 20 breath/min) and blood pressure (mean systolic pressure 66 – 100 mmHg). In keeping with the clinical history (suspected COVID-19), the patient had a moderately elevated temperature (39-40 ^o^C) on admission. At the time of physical examination, the patient was not distressed and did not require any supplemental oxygen therapy (inspired oxygen concentration was normal at 21%).

*Laboratory findings*

The patient was test positive for SARS-CoV-2. The leucocyte count on admission was normal, although the neutrophil-lymphocyte ratio was mildly abnormal (4.5). Otherwise, there was no other abnormal laboratory findings.

*IFI27 level*

The *IFI27* level on presentation was abnormal and extremely high (**707**).

*Clinical course*

The patient began to show signs of hypoxaemia on the second day after admission, with an increased respiratory rate (21-30 breath/min) and required supplemental oxygen (inspired oxygen concentration at 22%-29%). A further deterioration occurred on the third day where his inspired oxygen requirement increased to 30%-49%. On the fourth day, he developed hypoxic respiratory failure and was admitted to intensive care unit for mechanical ventilation.

*Summary*

Abnormal *IFI27* biomarker level precedes signs of respiratory failure by at least 24 hours.

**Patient B**

*History and physical examination*

This patient had no abnormal signs or symptoms on presentation, except for the presence of a fever (temperature 38.0-39.0 ^o^C). The pulse oximetry saturation reading was a bit on the low side; but it was still within the normal range (90-95% saturation while breathing at room air).

*Laboratory findings*

The patient was test positive for SARS-CoV-2. Laboratory findings showed mildly elevated C-reactive protein (CRP) levels on day 6 (CRP:12) and on day 7 (CRP:22), and neutrophil-lymphocyte ratio (4.5). At the time, these findings did not warrant any concern.

*IFI27 level*

However, the *IFI27* level on the same day was highly abnormal (**446**).

*Clinical course*

The patient become tachypnoeic two days later, with respiratory rate increased to 21-30 breath/min. He was admitted to hospital and given supplementary oxygen therapy. On the fourth day after admission, he developed hypoxic respiratory failure and was admitted to intensive care unit for mechanical ventilation. It was noted, on the day of admission to the intensive care unit, his neutrophil-lymphocyte ratio rose sharply to a highly abnormal level (6.8). A repeated *IFI27* on the same day also showed an abnormal result (**459**).

*Summary*

This *IFI27* warning sign preceded changes in bedside measurement or laboratory findings by several days. The *IFI27* level was already abnormally elevated prior to admission to intensive care unit - indicating the patient had an early sign of disease progression, despite the fact the vital signs and other bedside parameters were relatively “normal” at the time.

**Patient C**

*History and physical examination*

This patient presented with a high fever and cough. At the time of assessment, he had no abnormal physical signs and had no evidence of hypoxaemia.

*Laboratory findings*

The patient was test positive for SARS-CoV-2. Laboratory findings showed a mildly elevated C-reactive protein level (**16**), which did not warrant any concern.

*IFI27 level*

The *IFI27* level on the admission day was moderately abnormal (**226**).

*Clinical course*

The patient was admitted to hospital for monitoring because of positive SARS-CoV-2 status. The patient remained stable for the following three days and showed no signs of distress or hypoxaemia. However, a repeated *IFI27* measurement on the second day after admission revealed a rapidly rising *IFI27* level (**672**), which suggested an increasing risk of deterioration. On the fourth day, the patient suddenly developed severe hypoxic respiratory failure which required urgent admission to intensive care unit. On the same day, a further rise in *IFI27* level was noted (**881**), as was a highly abnormal C-reactive protein level (247 mg/L). Interestingly, on the day of deterioration, both leukocyte count and neutrophil-lymphocyte ratio remained within the normal range.

*Summary*

In this patient, the rise in *IFI27* levels occurred prior to clinical deterioration. Again, laboratory findings and changes in vital signs lagged behind *IFI27* rise.

**References**

1. Kulasinghe A, Tan CW, dos Santos Miggiolaro AFR, Monkman J, Bhuva D, Junior JdSM, et al. Spatial Profiling of Lung SARS-CoV-2 and Influenza Virus Infection Dissects Virus-Specific Host Responses and Gene Signatures. medRxiv. 2020.

2. Kicic A, Hallstrand TS, Sutanto EN, Stevens PT, Kobor MS, Taplin C, et al. Decreased fibronectin production significantly contributes to dysregulated repair of asthmatic epithelium. Am J Respir Crit Care Med. 2010;181(9):889-98.

3. Kicic A, Stevens PT, Sutanto EN, Kicic-Starcevich E, Ling KM, Looi K, et al. Impaired airway epithelial cell responses from children with asthma to rhinoviral infection. Clin Exp Allergy. 2016;46(11):1441-55.

4. Spann KM, Baturcam E, Schagen J, Jones C, Straub CP, Preston FM, et al. Viral and host factors determine innate immune responses in airway epithelial cells from children with wheeze and atopy. Thorax. 2014;69(10):918-25.

5. Gordon DE, Jang GM, Bouhaddou M, Xu J, Obernier K, White KM, et al. A SARS-CoV-2 protein interaction map reveals targets for drug repurposing. Nature. 2020;583(7816):459-68.
