# Supplementary material for: IFI27 transcription is an early predictor for COVID-19 outcomes, a multi-cohort observational study
Source: Front Immunol. 2023 Jan 5;13:1060438. doi: 10.3389/fimmu.2022.1060438 (PMC9850159; doi:10.3389/fimmu.2022.1060438)
Supplement: Supplementary file 4 [file Table_1.docx]

**Supplementary Table 1** Simplified version of the CDC definition of COVID-19 disease severity

| **COVID-19 related**  **Symptoms** | **ISARIC*-defined COVID 19**  **complications** |
| --- | --- |
|  |  |
| Fever | Viral pneumonia |
| Cough | Bacterial pneumonia |
| Sore throat | ARDS |
| Runny nose | Cardiac complications |
| Myalgia | Bacteraemia |
| Arthralgia | DIC |
| Fatigue | Liver dysfunction |
| Chest pain | Stroke/CVA |
| Shortness of breath | Seizure |
| Headache | Acute kidney injury |
| GITsymptoms (e.g., diarrhoea) | Hyperglycaemia |
| Loss of smell | Pulmonary embolism |
| Loss of taste | Deep venous thrombosis |
|  |  |

***ISARIC** denotes International Severe Acute Respiratory and Emerging Infection Consortium.
